# Asynchronous bilateral anastomosis site sigmoid colon cancer after ureterosigmoidostomy: a case report

**DOI:** 10.1186/s12957-016-0934-1

**Published:** 2016-07-07

**Authors:** Keiichi Arakawa, Soichiro Ishihara, Kazushige Kawai, Junichi Shibata, Kensuke Otani, Koji Yasuda, Takeshi Nishikawa, Toshiaki Tanaka, Tomomichi Kiyomatsu, Keisuke Hata, Hiroaki Nozawa, Hironori Yamaguchi, Eiji Sunami, Joji Kitayama, Toshiaki Watanabe

**Affiliations:** Department of Surgical Oncology, University of Tokyo, 7-3-1 Hongo, Bunkyo-ku, Tokyo, 113-8655 Japan

**Keywords:** Ureterosigmoidostomy, Sigmoidectomy, Ureterectomy, Adenocarcinoma, Colon cancer

## Abstract

**Background:**

We present a case of asynchronously occurring adenocarcinomas 29 and 36 years after ureterosigmoidostomy for bladder cancer, respectively, at both anastomosis sites.

**Case presentation:**

A colonoscopy that was performed on a 69-year-old man because of bloody stool and an elevated carcinoembryonic antigen (CEA) level revealed a polypoid lesion at the right ureterosigmoid anastomosis site 29 years after the patient’s ureterosigmoidostomy. Endoscopic resection was performed, and the lesion was diagnosed as adenocarcinoma. Seven years later (36 years after ureterosigmoidostomy), an elevated lesion was detected at the left ureterosigmoid anastomosis site by colonoscopy performed after detection of high CEA levels. Biopsy revealed an adenocarcinoma that was immunohistologically positive for CDX2; sigmoidectomy and ureterectomy were subsequently performed. The pathological diagnosis of the second tumor was adenocarcinoma arising in the ureterosigmoid anastomosis site and invading the left ureter.

**Conclusions:**

Diligent long-term follow-up of patients who underwent ureterosigmoidostomy is essential.

## Background

Ureterosigmoidostomy used to be commonly performed to treat bladder cancer. However, the procedure was recently abandoned because of complications such as hyperchloremia, renal failure, urinary infection, and adenocarcinoma at the ureterosigmoid anastomosis site. We herein discuss a rare case of adenocarcinoma occurring at both ureterosigmoid anastomosis sites; one was detected 29 years after the procedure while the other was detected 36 years afterwards.

## Case presentation

**Fig. 1:**
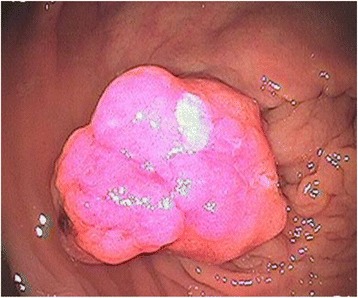
Intramucosal adenocarcinoma on the right anastomosis site of the ureterosigmoidostomy. Endoscopic mucosal resection was performed

A man underwent total cystectomy and ureterosigmoidostomy at 40 years of age to treat his bladder cancer. When he was 69 years old, he presented to our hospital with bloody stools and elevated tumor marker levels (carcinoembryonic antigen (CEA), 9.7 ng/mL). An elevated lesion at the right anastomosis site of the ureterosigmoidostomy was detected by colonoscopy (Fig. 1). Endoscopic resection was performed whereupon tumor marker levels decreased (CEA, 6.8 ng/mL). The pathological diagnosis was intramucosal adenocarcinoma. After resection, follow-up examinations were conducted using computed tomography (CT) and endoscopy. For 6 years, the tumor marker levels remained unchanged, and there was no evidence of recurrence.

**Fig. 2:**
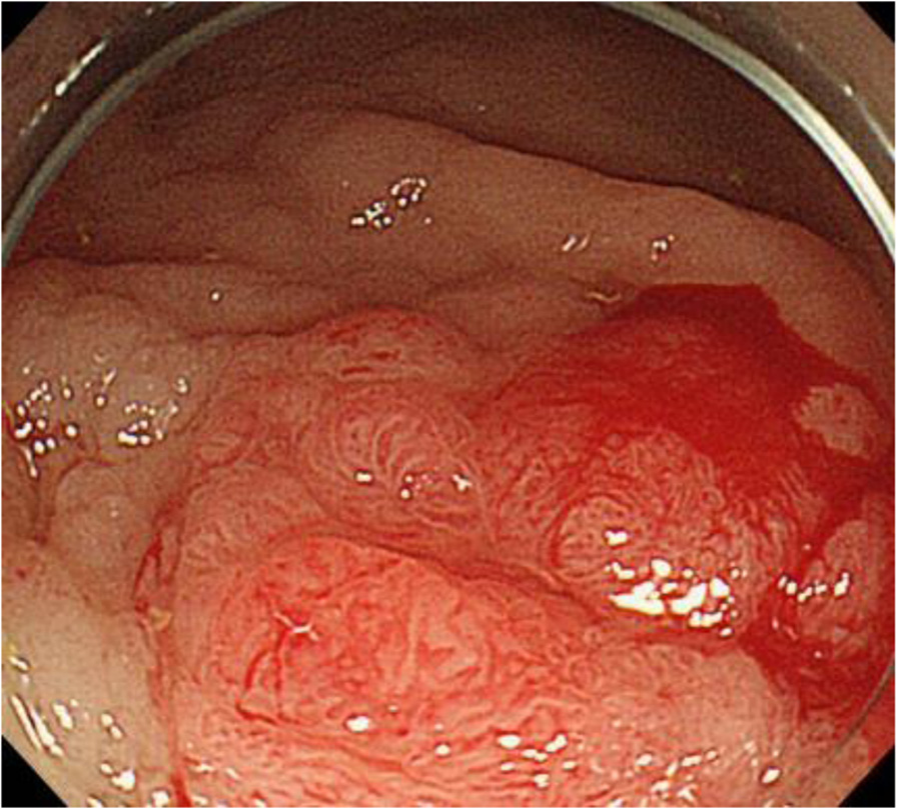
Submucosal adenocarcinoma on the left anastomosis site of the ureterosigmoidostomy. Sigmoidectomy and ureterectomy with a ureterocutaneous fistula were performed

**Fig. 3:**
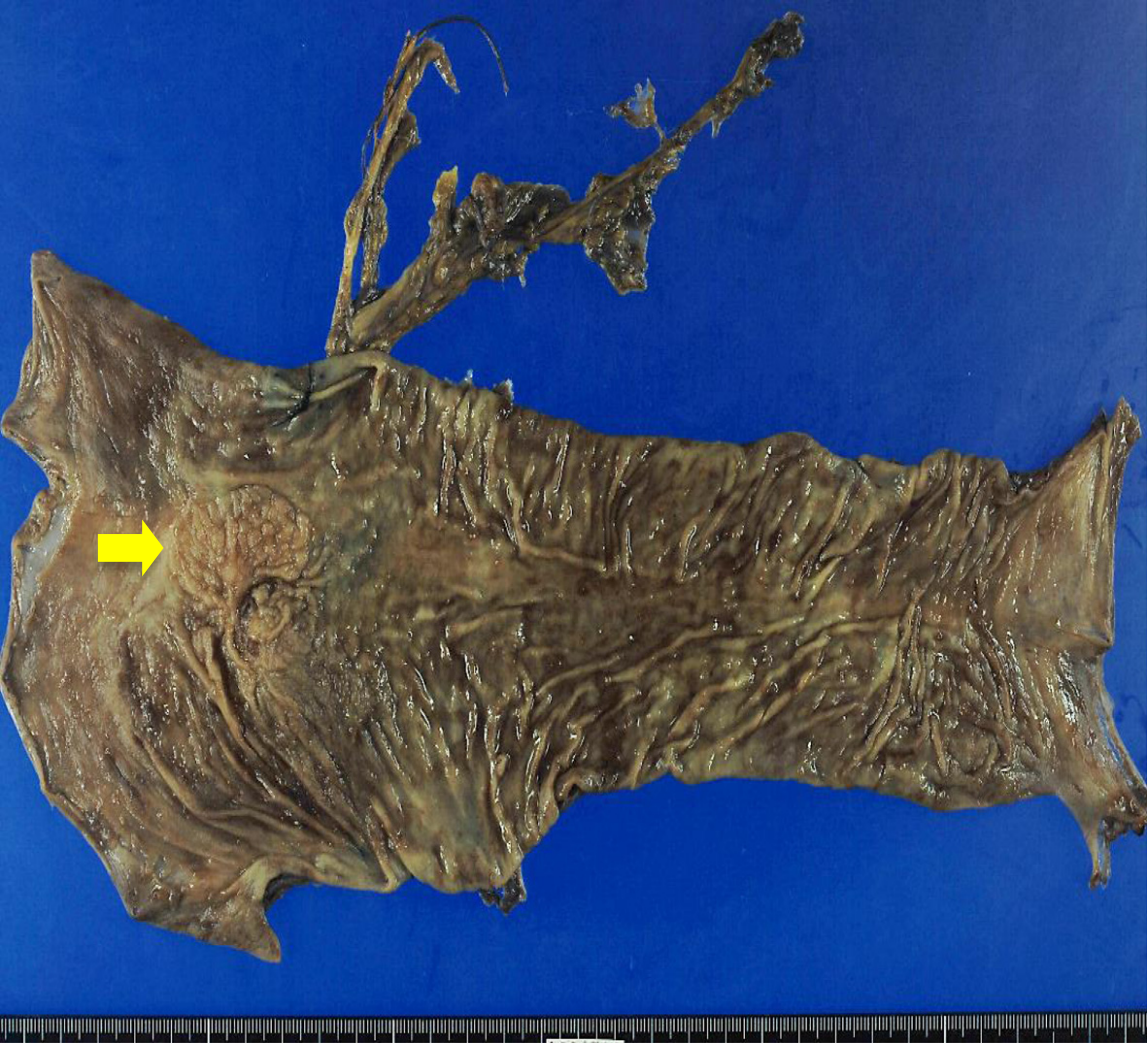
Macroscopic appearance of the resected specimen. Elevated lesions at the ureterosigmoid anastomosis site can be observed

**Fig. 4:**
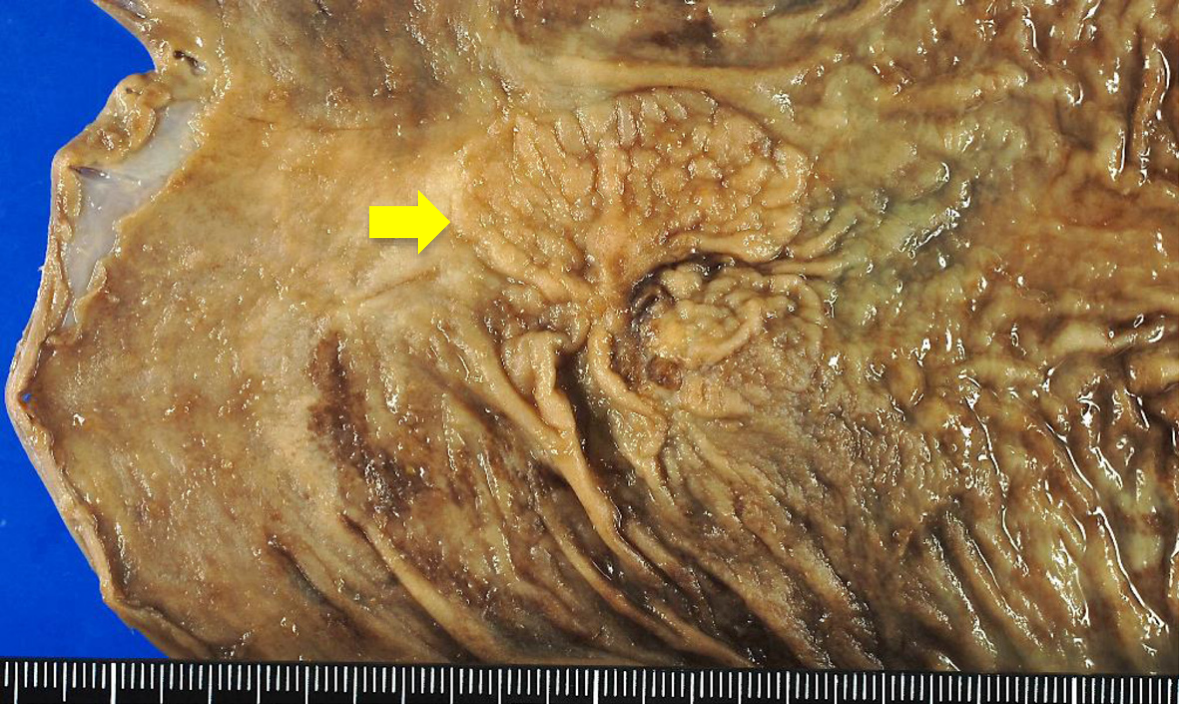
The tumor at the anastomosis site. The tumor measured 55 × 32 mm in size

**Fig. 5:**
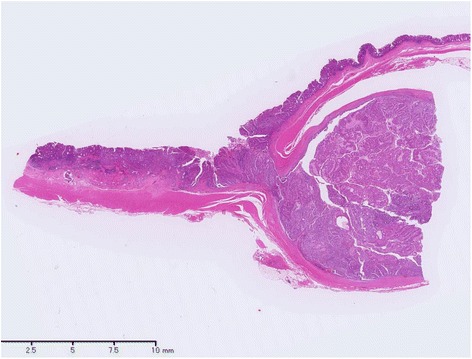
Microscopic findings (hematoxylin and eosin staining). Tumor tissue at the ureterosigmoid anastomosis site can be observed

**Fig. 6:**
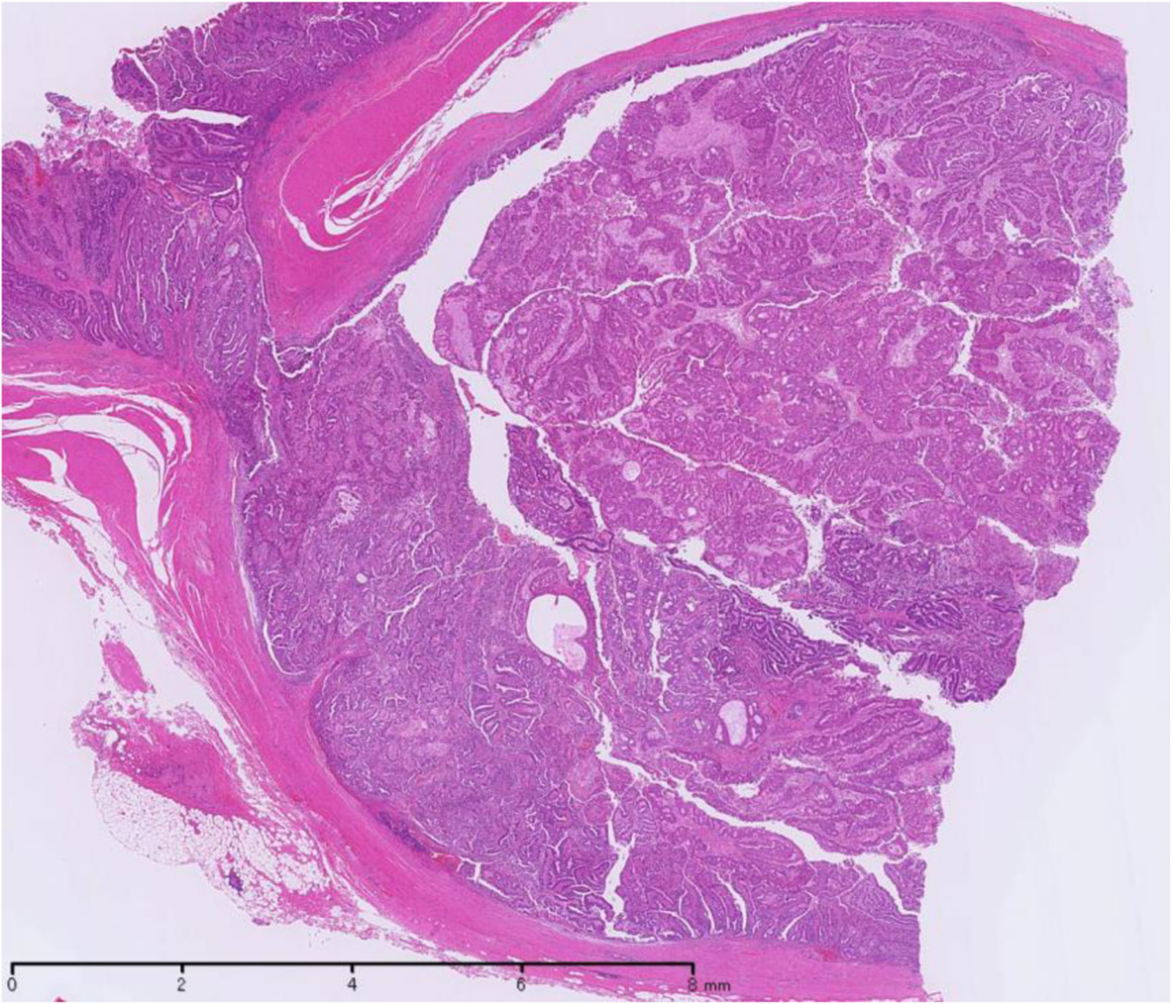
The left ureteral lumen was filled with tumor tissue

During a follow-up visit 7 years later, the patient’s CEA level was found to be elevated (25.3 ng/mL), and CT revealed a mass lesion at the left anastomosis site of the ureterosigmoidostomy. On admission, his breathing was deep and continuous (Kussmaul rhythm). Blood tests indicated renal dysfunction, acidosis, and hyperchloremia (creatinine: 1.72 mg/dL; pH: 7.252; chloride: 112 mEq/L); this was thought to be caused by the prior ureterosigmoidostomy and re-absorption of urine into the colon. The patient had bilateral hydronephrosis after ureterosigmoidostomy, predominantly on the right side, that had progressively worsened each year. His right renal function was additionally compromised after the endoscopic resection 7 years prior, and kidney function had been monitored during follow-up hospital visits. The renal failure upon admission was attributed to urinary flow obstruction by the tumor. On emergency admission, left nephrostomy was performed, which improved the renal dysfunction and acidosis. In addition to the mass lesion at the left anastomosis site, CT also revealed left pyelectasis. Colonoscopy detected an elevated lesion at the left anastomosis site, and biopsy revealed well-differentiated tubular adenocarcinoma and papillary adenocarcinoma (Fig. 2). Immunostaining was CDX2-positive, indicating adenocarcinoma from the intestinal epithelium. Thus, we diagnosed the patient with sigmoid colon cancer at the left ureterosigmoid anastomosis site. Sigmoidectomy and ureterectomy with an ureterocutaneous fistula were performed (Figs. 3 and 4). Pathology revealed adenocarcinoma in the elevated lesion at the ureterosigmoid anastomosis site and left ureter (Figs. 5 and 6). The patient required an extended hospital stay because of a postoperative catheter infection and ileus that required 3 weeks of treatment. After the 27^th^ postoperative day, kidney function was improved, and the indwelling ureteral stent was removed. The patient was discharged on the 32^nd^ postoperative day.

### Discussion

Ureterosigmoidostomy is a valid method for urinary diversion, as it is not associated with urinary incontinence. However, as exemplified by our patient, it is associated with urinary infection, renal dysfunction, acidosis from hyperchloremia, and colon cancer. In general, cancers that develop at the anastomosis site of a ureterosigmoidostomy tend to be one-sided. The patient in this case developed cancer at both anastomosis sites, which is rare. As such, this case highlights the importance of careful follow-up examinations after ureterosigmoidostomy. Because the pathological margin of the endoscopic resection was negative in our patient, the tumor at the left anastomosis site was deemed to have developed independently. The colon cancer was almost fatal for this patient.

Carcinoma at the site of the ureterocolic anastomosis was first reported in 1929 by Hammer [1]. Kälble et al. reported that the occurrence of colon cancer in patients who had undergone ureterosigmoidostomy was eight to ten times higher than that in the general population [2]. Urdaneta et al. calculated that the incidence of carcinoma after ureterosigmoidostomy was 13,300 per 100,000 cases [3]. The mean lag period for the development of colon carcinoma after surgery was 8.7 and 21.4 years for patients who were over and under the age of 40 years at the time of the operation, respectively [4]. The lesions were reported to be distal to the ureterosigmoidostomy site or involved the ureterocolonic junction [5]. Histological evaluations revealed the growth to be malignant twice as often as it was benign, with adenocarcinoma of the colon being the most frequent diagnosis [6].

The treatment for such tumors has changed over time. In the 1970s, sigmoid colon cancer arising at anastomosis sites after ureterosigmoidostomy was managed by local extirpation of the lesion, with re-implantation of one or both ureters back into the sigmoid. Currently, the preferred treatment is either endoscopic resection or more extensive surgical resection of the involved bowel segment combined with cutaneous loop diversions for an obvious malignant lesion. In this case, the patient opted for endoscopic therapy, and the right-sided tumor at the anastomosis site was completely resected endoscopically. However, the left-sided tumor was suspected of being an infiltrating cancer; therefore, surgical resection was performed. Mortality from this type of carcinoma is reportedly 33 % in cases of late detection that exhibit prominent metastases to regional lymph nodes [6].

One theory to explain the carcinogenesis of colonic carcinoma after ureterosigmoidostomy is the formation of nitrosamines from bacterially reduced urinary nitrates and from endogenous amines in feces and urine [2, 7–9]. Another theory is the urine-stool admixture concept, which suggests that hydrolytic enzymes in the urine activate conjugated carcinogens in the stool. These carcinogens are most active at the junction of the two streams where the greatest concentration exists [5, 10, 11]. Other factors, such as chronic irritation of the bowel epithelium, instability of the borderline between the urothelium and colonic epithelium, metabolic changes, and the presence of a fresh colonic suture line may contribute to the induction of colon carcinoma after ureterosigmoidostomy [2, 10–13]. Any one of these factors may be responsible for tumor development in our patient. To detect cancer before it attains advanced stage, surveillance colonoscopy should be performed at regular intervals after ureterosigmoidostomy because cancer or polyps can develop as early as 2 years after surgery and progress over a few decades [6]. The present case highlights the risk of cancer development after ureterosigmoidostomy as well as the importance of surveillance for early tumors. Based on the mechanism of tumorigenesis after ureterosigmoidostomy, tumors can develop either on the same or opposite side after resection, as in this case. Thus, follow-up examinations should be performed judiciously and at short intervals.

## Conclusions

We presented a case of adenocarcinomas occurring at both anastomosis sites after ureterosigmoidostomy that were detected 29 and 36 years after the original surgery, respectively. Our experience suggests that adenocarcinoma at both anastomosis sites can occur asynchronously; therefore, long-term attentive follow-up is necessary.

## Abbreviations

CEA, carcinoembryonic antigen; CT, computed tomography
